# Alterations of CSF Cystatin C Levels and Their Correlations with CSF Αβ40 and Αβ42 Levels in Patients with Alzheimer's Disease, Dementia with Lewy Bodies and the Atrophic Form of General Paresis

**DOI:** 10.1371/journal.pone.0055328

**Published:** 2013-01-29

**Authors:** Xiao-Mei Zhong, Le Hou, Xin-Ni Luo, Hai-Shan Shi, Guo-Yan Hu, Hong-Bo He, Xin-Ru Chen, Dong Zheng, Yue-Feng Zhang, Yan Tan, Xue-Jun Liu, Nan Mu, Jian-Ping Chen, Yu-Ping Ning

**Affiliations:** 1 Department of Neurology, Guangzhou Brain Hospital, Affiliated Hospital of Guangzhou Medical College, Guangzhou, People's Republic of China; 2 Department of Medical Laboratory, Guangzhou Brain Hospital, Affiliated Hospital of Guangzhou Medical College, Guangzhou, People's Republic of China; 3 Laboratory of Molecular Biology, Guangzhou Brain Hospital, Affiliated Hospital of Guangzhou Medical College, Guangzhou, People's Republic of China; 4 Department of Geriatric Psychiatry, Guangzhou Brain Hospital, Affiliated Hospital of Guangzhou Medical College, Guangzhou, People's Republic of China; University of Nebraska Medical Center, United States of America

## Abstract

Immunohistochemical studies have revealed that cystatin C (CysC) co-localizes with amyloid-β (Αβ) in amyloid-laden vascular walls and in the senile plaque cores of amyloid. In vitro and in vivo animal studies suggest that CysC protects against neurodegeneration by inhibition of cysteine proteases, inhibition of Αβ aggregation, induction of autophagy and induction of cell division. CysC levels may be altered and may have a potential link with cerebrospinal fluid (CSF) Aβ levels in various types of dementia with characteristic amyloid deposits, such as Alzheimer's disease (AD), dementia with Lewy bodies (DLB) and the atrophic form of general paresis (AF-GP). We assessed the serum and CSF levels of CysC and the CSF levels of Aβ40 and Aβ42 in patients with AD (n = 51), DLB (n = 26) and AF-GP (n = 43) and normal controls (n = 30). Using these samples, we explored the correlation between CSF CysC and CSF Aβ levels. We found that in comparison to the normal control group, both CSF CysC and CSF Aβ42 levels were significantly lower in all three dementia groups (all p<0.001); serum CysC levels were the same in the AD and DLB groups, and were lower in the AF-GP group (p = 0.008). The CSF CysC levels were positively correlated with both the CSF Aβ40 and Aβ42 levels in the AD, AF-GP and normal control groups (r = 0.306∼0.657, all p<0.05). Lower CSF CysC levels might be a common feature in dementia with characteristic amyloid deposits. Our results provide evidence for the potential role of CysC involvement in Aβ metabolism and suggest that modulation of the CysC level in the brain might produce a disease-modifying effect in dementia with characteristic amyloid deposits.

## Introduction

Cystatin C (CysC) is secreted by all human cells and found in all mammalian body fluids and tissues. It is almost completely cleared from the circulation by glomerular ultrafiltration [1]. CysC is involved in many pathological processes, including oxidative stress, neurodegeneration and inflammation [1], [2]. A growing number of studies have demonstrated that CysC plays important protective roles in neurodegenerative diseases via various mechanisms [3], [4]. In vitro and in vivo animal studies show that CysC plays an ongoing role in the inhibition of amyloid-β (Αβ) oligomerization and fibril formation by binding both Αβ40 and Αβ42 in a specific, saturable and high-affinity manner [5], [6]. Overexpression of CysC decreases cathepsin B and D activities and rescues degenerating neurons in the brain of cystatin B knockout mice which were shown to have an increased cathepsins activity [7]. CysC can also regulate cell proliferation and migration [8]. Furthermore, CysC induces a fully functional autophagy via the mTOR pathway in cells under basal conditions, and enhances the autophagic activation in cells exposed to nutritional deprivation and oxidative stress [9].

CysC has been found to co-localize with Aβ in the senile plaque cores of amyloid plaques in brains of patients with amyloidosis [2], [10]–[13]. Alzheimer's disease (AD) is the most common form of neurodegenerative dementia with characteristic amyloid deposits. Aβ accumulation in the brain, which leads to oxidative and inflammatory damage, is considered to be a critical event in AD pathology [14]. Dementia with Lewy bodies (DLB), the second most common form of neurodegenerative dementia, has neuropathological changes characteristic of AD, in particular amyloid plaques. Approximately 70%∼80% of DLB patients exhibit amyloid pathology in the brain [15], [16]. In addition to AD and DLB, general paresis (GP), a chronic meningoencephalitis caused by direct invasion of the brain parenchyma by spirochetes, is also associated with amyloid deposits [17]. Patients with GP usually present with atrophy of the medial temporal lobe and AD-like cognitive impairment [18]. Inflammation and neurodegeneration can be found in the cortex of patients with GP [19]. Observations on the distribution of spirochetes in the GP brain suggest the occurrence of two different forms of GP, the infiltrative and atrophic forms [17]. It has been demonstrated that the atrophic form of general paresis (AF-GP) is associated with slowly progressive dementia, brain atrophy, oxidative stress, chronic inflammation, neurofibrillary tangles and amyloid deposits [17]. As in AD, amyloid deposits in GP comprise Aβ [20].

AD, DLB and AF-GP share similar pathological process of neurodegeneration with amyloid deposits, oxidative stress and inflammation. Experimental [5], [6], genetic [21]–[23] and clinical [10], [24], [25] data have suggested that CysC protests against the development of AD. Alterations of cerebrospinal fluid (CSF) CysC levels in patients with AD have been documented, but the results are contradictory [26]–[31]. Given the suggested involvement of CysC in amyloid deposits and neurodegeneration, we hypothesized that CysC levels may be altered and linked with CSF Aβ levels in a variety of dementia with characteristic amyloid deposits. In this study, we therefore assessed the levels of CysC in serum and CSF and the levels of Aβ40 and Aβ42 in CSF in patients suffering from AD, DLB and AF-GP, as well as in normal controls. We also examined the potential link between the CSF levels of CysC and Αβ.

## Materials and Methods

### Ethics statement

All subjects gave their written informed consent. In case patients were considered not to have the capacity, written inform consent was given by the nearest relatives. The study was conducted according to the principles expressed in the Declaration of Helsinki and approved by the ethics committees at the Guangzhou Brain Hospital.

### Subjects

A total of 150 subjects (51 AD, 26 DLB, 43 AF-GP, 30 normal controls) were consecutively recruited from the ward and the outpatient clinic of the Neurology Department of the Guangzhou Brain Hospital. Both a neurologist and a psychiatrist evaluated all of the individuals. All enrolled patients fulfilled the Diagnostic and Statistical Manual of Mental Disorders-IV (DSM-IV) criteria for dementia. The AD patients fulfilled the NINCDS-ADRDA [32] criteria for the clinical diagnosis of probable AD. The DLB patients fulfilled the McKeith criteria [33] for the clinical diagnosis of probable DLB. Currently, there is no gold standard for clinical diagnosis of GP. Its diagnosis is based on a combination of clinical evaluation, serologic testing and CSF examination [17], [34]. In our study, the diagnosis of GP was based on appropriate clinical features (including personality changes, decreased language ability, decreased motivation, impaired judgment, loss of ability to calculate, loss of long-term memory, loss of short-term memory) and positive results of T. pallidum hemagglutination assay reactions in blood and CSF samples. GP patients who met the following criteria were allocated to the category of AF-GP: a slowly progressive dementia and a diffuse cortical atrophy as shown by CT or MRI [17], [34]. The normal controls were defined as subjects with no neurological or psychiatric conditions. All subjects underwent interview, neuropsychological assessment, neuroimaging and appropriate laboratory tests including vitamin B12, folate and thyroid hormone status to confirm the accuracy of the clinical diagnosis.

### Serum and CSF collection

The CSF samples (10–12 ml) were collected in the morning by lumbar puncture. The first 2 ml of CSF was examined for cell counts, glucose and total protein. The CSF samples with erythrocyte counts less than 500 cells/µL were required for further analysis. Within 1 hour after lumbar puncture, blood samples (12–15 ml) were collected. All of the samples were centrifuged at 3000× g for 15 minutes at 4°C after collection. The CSF and serum were aliquoted into polypropylene tubes and stored at −80°C until use.

### Serum and CSF measurement

The serum and CSF CysC levels were quantitatively measured by a latex immunoturbidimetric assay using CysC reagents (Dako, Denmark) with an auto-analyzer (Architect C8000; Abbott Laboratories, Abbott Park, IL, USA) with the following instrument settings: Wavelength: 546 nm, 2 μL samples and 160 μL reagent 1 were mixed with 40 μL reagent 2. The CSF Αβ40 and Αβ42 levels were quantified using commercially available enzyme immunoassay kits (Innogenetics, Belgium). All the samples were analyzed in duplicate.

### Assessment of glomerular filtration rate (GFR)

We used the Modification of Diet in Renal Disease 4 equation (MDRD4, GFR  = 186.3×Serum Cr^−1.154^×age^−0.203^×1.212 (if patient is black) ×0.742 (if female)) to estimate the GFR for each participant. The serum levels of creatinine were analyzed using a standard technique.

### Statistical analysis

The statistical analysis was performed with the SPSS (Statistical Package for the Social Sciences) 13.0 statistical software. The Kolmogorov-Smirnov test was performed for all of the continuous variables to define the presence of normality. Comparisons of normally distributed data were performed using a one-way analysis of variance (ANOVA), followed by post hoc Bonferroni's test. When comparing CysC levels, analysis of covariance (ANCOVA) was also used to adjust for potential confounders for association with CysC, including age and GFR. Non-parametric analysis was performed for non-normally distributed data and categorical variable. The correlations of the measured values were examined using the Pearson correlation coefficient. A p value of less than 0.05 was considered statistically significant.

## Results

### Demographic and clinical characteristics

There were no significant differences in education among the four groups. We found a lower percentage of female patients (p<0.001) and younger ages (p<0.001) in the AF-GP group compared to the normal control group (Table 1). As expected, all three dementia groups (AD, DLB and AF-GP) had significantly higher scores on the ADAS-cog and lower scores on the MMSE and MoCA compared to the normal control group (all p<0.001) (Table 2). The Geriatric Depression Scale (GDS) scores were significantly higher in the DLB (p<0.001) and AF-GP (p<0.001) groups compared with the normal control group (Table 2). No significant differences in GFR were found among the four groups.

**Table 1:** Demographic characteristics.

	AD	DLB	AF-GP	NC
No.	51	26	43	30
Ethnic origin	Chinese Han	Chinese Han	Chinese Han	Chinese Han
Female gender (%)	58.8%	61.5%	20.9%***	63.3%
Age (years)	68.5±8.7	68.4±9.8	51.4±11.7***	64.3±7.1
Education (years)	8.4±3.5	10.1±3.3	9.5±2.7	8.5±2.3

*** p<0.001 compared with normal controls.

Abbreviations: AD, Alzheimer's disease; DLB, dementia with Lewy body; AF-GP, the atrophic form of general paresis; NC, normal control.

**Table 2:** Clinical data.

	AD	DLB	AF-GP	NC
No.	51	26	43	30
MMSE	11.1±5.8***	13.8±5.7***	17.8±5.7***	28.0±1.2
MoCA	6.9±5.5***	7.4±4.5***	13.2±5.8***	26.8±0.9
ADAS-cog	46.5±13.2***	41.0±14.8***	31.1±12.3***	7.0±2.1
GDS	2.3±1.2	7.2±3.3***	3.4±2.8***	1.3±0.9
NPI total	18.0±11.7	24.8±9.6	17.8±13.2	na
CDR Staging (0/1/2/3)	0/8/17/26***	0/4/17/5***	0/16/22/5***	30/0/0/0
GFR (ml/min)	98.7±26.0	94.5±17.1	104.3±26.9	104.2±26.3

*** p<0.001 compared with normal controls.

Abbreviations: AD, Alzheimer's disease; DLB, dementia with Lewy body; AF-GP, the atrophic form of general paresis; NC, normal control; MMSE, Mini Mental State Examination; MoCA, Montreal Cognitive Assessment; ADAS-cog, Alzheimer's Disease Assessment Scale-cognitive subscale; GDS, Geriatric Depression Scale; NPI, Neuropsychiatric Inventory; CDR, Clinical Dementia Rating Scale; GFR, glomerular filtration rate; na, not applicable.

### CysC, Αβ40 and Αβ42 levels

**Figure 1:**
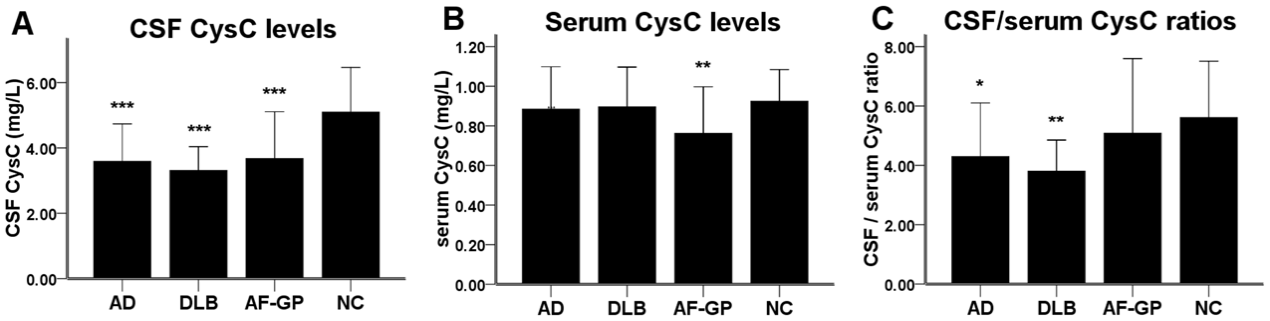
The CSF and serum CysC levels in AD, DLB, AF-GP and normal control subjects. (A) The levels of CSF CysC were significantly lower in patients with AD, DLB and AF-GP compared to normal controls. (B) The levels of serum CysC were significantly lower in patients with AF-GP compared to normal controls. (C) The CSF/serum CysC ratios were significantly lower in patients with AD and DLB compared to normal controls. Statistically significant difference was calculated using one-way ANOVA, followed by Bonferroni's post hoc test. Data are means ± SD. Abbreviations: CysC, cystatin C; AD, Alzheimer's disease; DLB, dementia with Lewy body; AF-GP, the atrophic form of general paresis; NC, normal control. (*p<0.05, **p<0.01, ***p<0.001).

**Figure 2:**
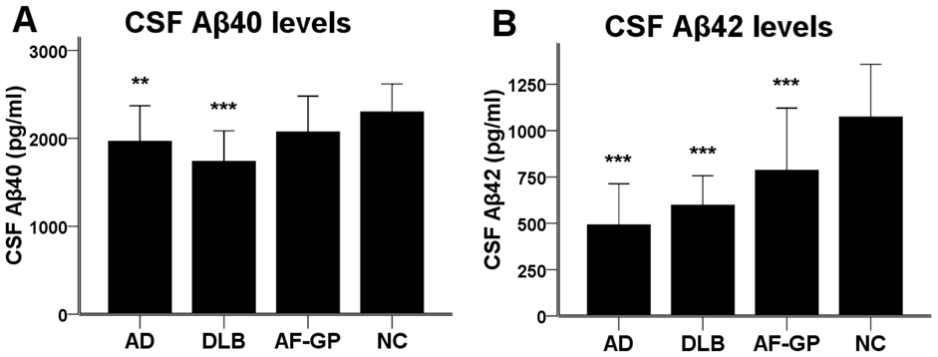
The CSF Αβ40 and Αβ42 levels in AD, DLB, AF-GP and normal control subjects. (A) The levels of CSFΑβ40 were significantly lower in patients with AD and DLB compared to normal controls. There was a trend towards decreased CSF Αβ40 levels in patients with AF-GP compared to normal controls (p = 0.075). (B) The levels of CSF Αβ42 were significantly lower in patients with AD, DLB and AF-GP compared to normal controls. Statistically significant difference was calculated using one-way ANOVA, followed by Bonferroni's post hoc test. Data are means ± SD. Abbreviations: Αβ, amyloid-β protein; AD, Alzheimer's disease; DLB, dementia with Lewy body; AF-GP, the atrophic form of general paresis; NC, normal control. (**p<0.01, ***p<0.001).

The CSF CysC levels were significantly lower in all three dementia groups compared to the normal control group (AD: p<0.001, DLB: p<0.001, AF-GP: p<0.001) (Fig. 1A) These differences remained significant (AD: p<0.001, DLB: p<0.001, AF-GP: p = 0.006) after adjusting for age and GFR. The serum CysC levels were significantly lower in the AF-GP group than in the normal control group (p = 0.008) (Fig. 1B). The difference remained robust (p = 0.007) after adjusting for age and GFR. The serum CysC levels were not different from the normal control group in patients with AD and DLB. The CSF/serum CysC ratios were significantly lower than normal controls in patients with AD (p = 0.024) and DLB (p = 0.004), but not in patients with AF-GP (p = 1.0) (Fig. 1C). Both the AD and DLB groups had lower CSF levels of Αβ40 (AD: p = 0.001, DLB: p<0.001) and Αβ42 (AD: p<0.001, DLB: p<0.001) compared to the normal control group (Fig. 2A and 2B). The AF-GP group displayed lower CSF Αβ42 levels (p<0.001) with a trend towards decreased CSF Αβ40 levels (p = 0.075), while compared with the normal control group (Fig. 2A and 2B).

### Correlations of measured values

**Figure 3:**
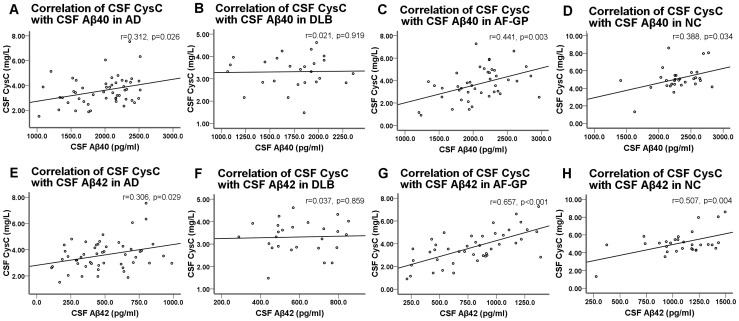
Scatter plots of the correlations between CSF CysC and CSF Αβ40 and Αβ42 levels. (A) (C) (D) The CSF CysC levels were positively correlated with CSF Αβ40 levels in AD (r = 0.312, p = 0.026), AF-GP (r = 0.441, p = 0.003) and normal control (r = 0.388, p = 0.034) subjects. (E) (G) (H) The CSF CysC levels were positively correlated with CSF Αβ42 levels in AD (r = 0.306, p = 0.029), AF-GP (r = 0.657, p<0.001) and normal control (r = 0.507, p = 0.004) subjects. (B) (F) No significant correlations were found between CSF CysC and CSF Αβ40 or Αβ42 in patients with DLB (r = 0.021, p = 0.919, and r = 0.037, p = 0.859, respectively). The correlations of the measured values were examined using the Pearson correlation coefficient. Abbreviations: CysC, cystatin C; Αβ, amyloid-β protein; AD, Alzheimer's disease; DLB, dementia with Lewy body; AF-GP, the atrophic form of general paresis; NC, normal control.

The serum CysC levels were negatively correlated with GFR (r = −0.279, p = 0.001) and positively correlated with age (r = 0.188, p = 0.021) in the total sample. There were no correlations between CSF CysC levels and GFR (r = 0.034, p = 0.68) or age (r = 0.129, p = 0.115). CSF CysC levels were positively correlated with serum CysC levels in the total sample (r = 0.186, p = 0.023). We also analyzed the correlation between CSF and serum levels of CysC according to the tertiles of CSF Αβ42 levels in the total sample. A significant positive correlation was found in subjects in the lowest CSF Αβ42 tertile (CSF Αβ42 level: 108.4 pg/ml ∼490.7 pg/ml) (r = 0.290, p = 0.041), but not in those within the middle (CSF Αβ42 level: 490.8 pg/ml ∼847.4 pg/ml) (r = 0.115, p = 0.428) and highest (CSF Αβ42 level: 847.5 pg/ml ∼1495.0 pg/ml) (r = 0.107, p = 0.460) tertiles. CSF CysC levels were positively correlated with CSF Αβ40 and Αβ42 levels in the AD, AF-GP and normal control groups (Table 3). However, no such correlations were found in the DLB group. Scatter plots of the correlations of CSF CysC with CSF Αβ40 and Αβ42 are presented in Figure 3.

**Table 3:** Correlations between CSF CysC and CSF Αβ40 and Αβ42 levels.

	AD (n = 51)		DLB (n = 26)		AF-GP (n = 43)		NC (n = 30)	
	r	p	r	p	r	p	r	p
Αβ40	0.312	0.026	0.021	0.919	0.441	0.003	0.388	0.034
Αβ42	0.306	0.029	0.037	0.859	0.657	<0.001	0.507	0.004

Abbreviations: AD, Alzheimer's disease; DLB, dementia with Lewy body; AF-GP, the atrophic form of general paresis; NC, normal control; CysC, cystatin C; Αβ, amyloid-β.

### Association of CysC levels with disease severity

**Figure 4:**
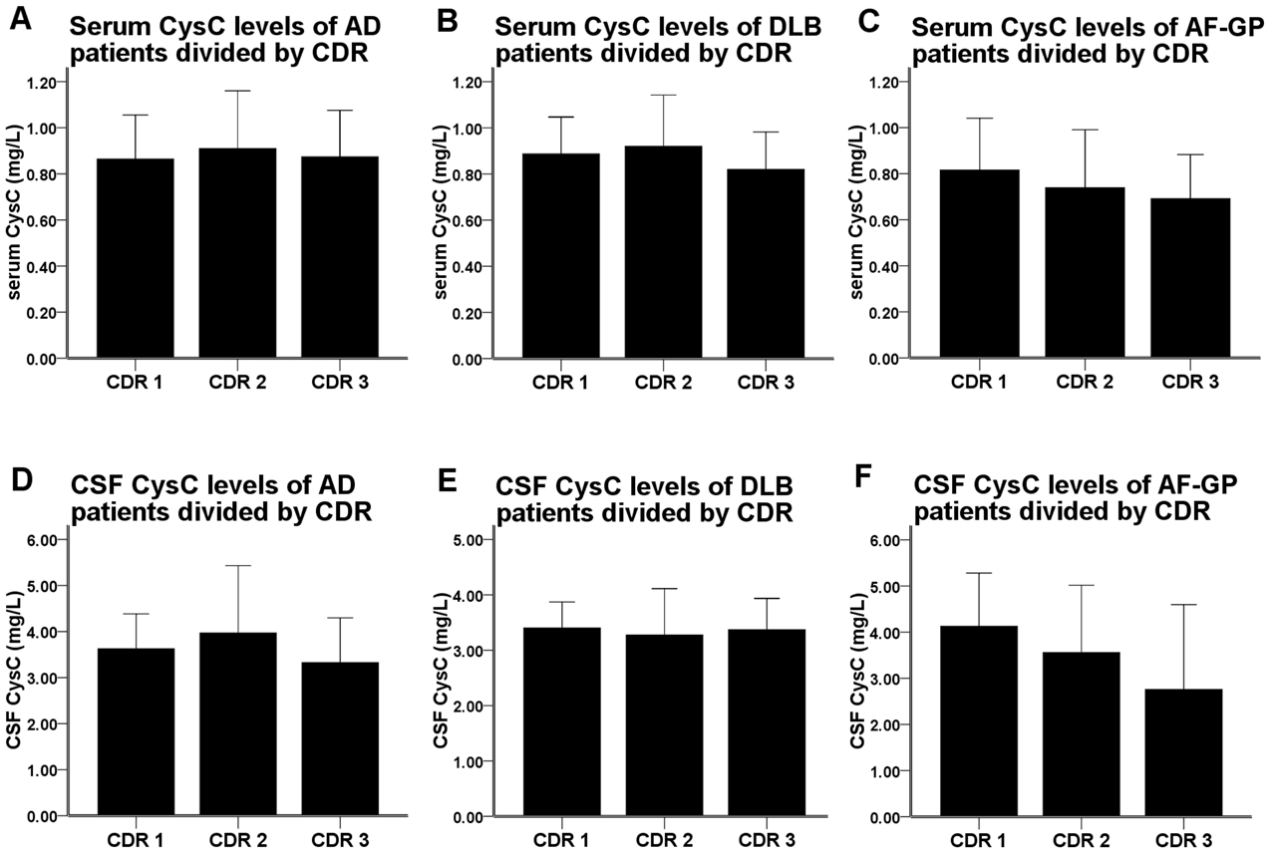
The serum and CSF CysC levels of dementia patients divided by CDR scores. No significant differences in serum or CSF CysC levels were observed in patients with AD (A and D), DLB (B and E) and AF-GP (C and F). Statistically significant difference was calculated using one-way ANOVA. Data are means ± SD. Abbreviations: CysC, cystatin C; CDR, Clinical Dementia Rating Scale; AD, Alzheimer's disease; DLB, dementia with Lewy body; AF-GP, the atrophic form of general paresis.

To explore the association between the CysC levels and the disease severity, we compared the serum and CSF CysC levels among groups defined by Clinical Dementia Rating Scale (CDR). Based on their CDR scores (CDR 1, CDR 2, and CDR 3), patients with AD, DLB and AF-GP were divided into three groups respectively. No significant differences in serum or CSF CysC levels were observed as determined by one-way ANOVA (Fig. 4) or by ANCOVA adjusting for age and GFR. We also correlated the serum and CSF levels of CysC with MMSE, MoCA and ADAS-cog scores. However, we failed to find any significant correlation.

## Discussion

To dig up biological evidence for a role of CysC in dementia with characteristic amyloid deposits, we evaluated the serum and CSF levels of CysC and the correlation of CSF CysC with CSF Aβ in patients with AD, DLB and AF-GP and normal controls. Our main finding was that the CSF CysC levels were significantly lower in all three dementia groups compared with normal controls. Moreover, positive correlations were detected between CSF CysC levels and CSF Aβ40 and Aβ42 levels in patients with AD and AF-GP, as well as in normal controls.

### CSF and serum CysC levels in dementia with characteristic amyloid deposits

In consistent with recent published data [26], [27], [30], [31], [35], we found lower levels of CSF CysC in patients with AD. Maetzler et al. reported decreased CSF CysC levels in demented Lewy body disease patients [26], [27], [35]. Similarly, we also observed reduced CSF CysC levels in patients with DLB. In addition, our data showed, for the first time to our knowledge, that patients with AF-GP had significantly lower CSF CysC levels. These results indicate that lower CSF CysC level is a common neurochemical feature in patients with AD, DLB and AF-GP. On the basis of these observations, we propose that decreased CSF CysC levels could serve as a potential marker for dementia with characteristic amyloid deposits. On the standard AD markers, our findings confirmed the results of decreased CSF Αβ42 levels in patients with AD. The most likely explanation for the decreased CSF Aβ42 in AD is that the Aβ peptides aggregate into plaques, resulting in the retention of Aβ in the brain parenchyma and less Aβ being available to diffuse into the CSF [36]. Lower levels of CSF Aβ42 in patients with DLB and AF-GP observed in our study reflect the presence of AD-like Aβ deposition in the brain. CysC was found to co-localize with Αβ predominantly in amyloid-laden vascular walls and in the senile plaque cores of amyloid in the brains of AD, Down syndrome, cerebral infarction, cerebral amyloid angiopathy and non-demented aged individuals [2], [10]–[13]. Decreased CSF CysC in patients with characteristic amyloid deposits may be related to its deposition occurring secondarily to Αβ deposition [13] in the brain. Several studies documented an association of the BB genotype of the CysC-encoding gene CST3 with decreased plasma [24] and CSF [35], [37] CysC levels, and this association may be due to a less efficient cleavage of the signal peptide, resulting in a reduced secretion of CysC [38]. These results, in combination with our data, suggest that decreased levels of CSF CysC in dementia patients with characteristic amyloid deposits may be explained by abnormal deposition in amyloid plaques in the brain, reduced secretion of the protein or both.

The serum CysC, which is cleared from the circulation by glomerular filtration, is affected by renal function [1]. The positive correlation between serum CysC and age noticed in this study could be attributable to age-related changes in renal function. Both One-way ANOVA and ANCOVA adjusting for age and GFR showed significantly lower levels of serum CysC in patients with AF-GP. Chronic syphilitic infections are frequently associated with amyloid deposition in the infected tissues, including the skin, mucous membranes, bones, joints and various internal organs [17]. CysC might accumulate in amyloid deposits in the tissues and organs of patients with AF-GP, resulting in a depletion of the serum levels. Of all the three dementia groups, normal CSF/serum ratios were noticed only in the AF-GP group. Simultaneous decrease of serum and CSF CysC levels in patients with AF-GP could contribute to these results.

### Evidences for the involvement of CysC in Aβ metabolism

Recent studies reported positive correlations of CSF CysC with CSF Aβ40 and Aβ42 in patients with AD and normal controls [26], [29], [30], [35]. We confirmed these findings in current study. Interestingly, such correlations were also observed in patients with AF-GP. CysC was demonstrated to be included at the ratio of about 1:100 in the crude amyloid fibrils [39] and inhibit Aβ aggregation and deposition in a concentration-dependent manner by binding to the Aβ40 and Aβ42 [5], [40]. These results might explain the correlation between CSF CysC and Aβ. Moreover, the detected positive correlations were not restricted to patients suffering from AD and AF-GP, but also existed in normal controls, indicating that CysC is involved in both pathological conditions of amyloid deposits and physiological conditions of normal aging, with consistent trend of Aβ. These correlations suggest an interaction of CysC with Aβ metabolism. However, no correlations between CSF CysC and either CSF Aβ40 or CSF Aβ42 were observed in patients with DLB, which were in accordance with the results shown in the study by Maetzler et al. [35]. It is possible that there are potential interference factors that break the correlations of CysC with Aβ in DLB brain. Further studies into these factors are needed.

Our results showed that CysC levels were lower only in the CSF of patients suffering from AD and DLB, but reduced in both the CSF and serum of patients suffering from AF-GP. Amyloid deposits are present in the brain of AD and DLB, while in GP, they accumulate both in the central nervous system and the periphery. It seems that there are decreased CysC levels in the tissues, organs or systems where there is a certain amount of amyloid deposits. These findings provide further support for the notion that CysC is related to Aβ metabolism.

### Possible impact of lower levels of CSF CysC

Several protective effects of CysC on the brain that plays a role in neurodegeneration have been described in in vitro and in vivo studies, ranging from inhibition of cysteine proteases [7], induction of autophagy [9], induction of neurogenesis [8] and anti-amyloidogenesis [5], [6]. Thus, lower levels of CSF CysC manifested in patients with AD, DLB and AF-GP may result in decreased ability to inhibit the activity of cathepsins, impaired cytoprotective response to prevent neurodegeneration in various pathological conditions, decreased level of neurogenesis and decreased ability to inhibit neuronal Αβ aggregation and deposition. Lower levels of CysC may contribute to both the pathology (amyloidogenic process) and the progression (resulting in increased neuronal vulnerability) of dementia with characteristic amyloid deposits. However, it is not possible to establish a causal role for CysC in the pathogenesis in our study.

### Utilities and future prospects of CysC

Relatively low level of serum or plasma CysC was supposed to be a marker for future risk of AD [25], [41]. The positive correlation between the serum and CSF levels of CysC observed in our total sample suggests that the serum CysC levels may partly reflect the CSF CysC levels. Therefore, person with a relatively low level of serum CysC might have a relatively low level of CSF CysC, a reduced ability to inhibit neuronal Αβ aggregation [25] and a high risk of developing AD. When stratified by CSF Aβ42 levels, the correlation between CSF and serum CysC levels was significant in subjects in the lowest CSF Aβ42 tertile, supporting the idea of the appearance of a pathological event that affects the serum CysC levels in subjects with severe Aβ deposition, with consistent trend of CSF CysC levels. Further studies are needed to explore this important issue. Our study assessed the CysC levels cross-sectionally; therefore, we were unable to investigate the relationship of CysC levels with cognitive changes. Longitudinal studies of CysC levels in normal aging and mild cognitive impairment are required to explore the association of CysC with disease progression, and to investigate whether CysC might be a preclinical marker of dementia with characteristic amyloid deposits.

Previous studies showed that the CysC levels in CSF of AD patients [26], [27], [30], [31], [35] were lower, while the levels of CysC were not changed from normal controls in CSF of frontotemporal dementia patients [27]. Thus, it was suggested that CysC could serve as a biomarker to improve the accuracy of the AD diagnosis [27], [30], [31]. In the current study, lower levels of CSF CysC are not a specific feature of AD, but also associated with DLB and AF-GP, indicating that CysC will not be useful in differential diagnosis among AD, DLB and AF-GP.

Decreased CSF Αβ42 levels had been reported to be associated with disease severity [42], [43]. We were able to correlate lower levels of CSF CysC to lower levels of CSF Αβ40 and Αβ42 in patients with AD and AF-GP. Therefore, we asked the question whether the CysC levels are associated with severity of dementia with characteristic amyloid deposits. We explored the correlations between CysC and global cognitive function assessed by MMSE, MoCA and ADAS-cog, and compared the different levels of CysC among CDR 1, CDR 2, and CDR 3. However, we did not find any association of CSF or serum CysC with either cognitive impairment or severity of symptoms of dementia in patients with AD, DLB and AF-GP. These findings do not support the clinical utility of CysC level as a marker of severity of dementia with characteristic amyloid deposits.

Although CysC has multi-targeted neuroprotection in the brain, its levels expression in patients with AD, DLB and AF-GP might not be sufficient to maintain the long-term inhibition of Αβ deposition and adequately protect the neuronal cells from Aβ toxicity and oxidative stress. It had been demonstrated that CysC protects neuronal cells from death in a concentration-dependent manner [9]. An increase in CysC of twofold above endogenous levels were sufficient to diminish Αβ deposition in the AD mouse model [6]. Thus, modulation of the CysC level in the brain or periphery, or mimicking its action, would be beneficial in preventing, slowing, or halting disease progression.

### Limitation of the study

A limitation of our study is the lack of amyloid plaque imaging in our subjects. Patients with pure DLB without any amyloid pathology may be included. However, there are strengths in this study. We analyze the CysC level in both the serum and CSF in different types of dementia with characteristic amyloid deposits. Furthermore, the causes of dementia include not only degenerative neurological diseases but also infections of the central nervous system.

## Conclusions

In summary, our results suggest that lower levels of CSF CysC are related to dementia with characteristic amyloid deposits. Decreased levels of Aβ42 are accompanied by a decrease in the CSF CysC levels in patients with AD, DLB and AF-GP, and there are positive correlations between these proteins in AD, AF-GP and normal control subjects. Our findings strengthen the evidence for the role of CysC involved in Aβ metabolism. Modulation of the CysC level may represent an alternative therapeutic strategy in dementia with characteristic amyloid deposits.
